# Epidemiological and antimicrobial susceptibility patterns of *Staphylococcus aureus* isolated from the nasal cavity of healthcare personnel at a public hospital in Northeastern Brazil: a cross-sectional survey

**DOI:** 10.1007/s42770-026-02126-0

**Published:** 2026-11-04

**Authors:** Francisco Italo Gomes Alencar, Francisco Marques da Silva Neto, Nayara Maria Barbosa de Sousa, Ana Alice Venancio Correia, Maria Anndressa Alves Agreles, José Carlos da Rocha Neto, Danilo Elias Xavier, Damilola Joseph Olaoye, Luís André de Almeida Campos, Francisco Laurindo da Silva, Isabella Macário Ferro Cavalcanti

**Affiliations:** 1 Laboratory of Microbiology and Immunology of Infectious and Parasitic Diseases (LAMIDI), Department of Health Sciences, University of Maranhão (UEMA), Caxias, Maranhão Brazil; 2 Department of Statistics, Federal University of Lavras (UFLA), Lavras, Minas Gerais Brazil; 3 Federal University of Pernambuco (UFPE), Keiso Asami Institute (iLIKA), Recife, Pernambuco Brazil; 4 Department of Microbiology, Aggeu Magalhães Institute, Oswaldo Cruz Foundation Recife, Pernambuco, Brazil; 5 Department of Microbiology, Awolowo University, Federal University of Pernambuco (UFPE), Keiso Asami Institute (iLIKA), Ile-Ife, Recife, Osun State, Pernambuco Nigeria Brazil; 6 Laboratory for Teaching, Research and Extension in Microbiology and Parasitology (LEPEMP), University of Pernambuco, Campus Ouricuri, Ouricuri, Pernambuco Brazil; 7 Federal University of Pernambuco (UFPE), Academic Center of Vitória (CAV) and Keiso Asami Institute (iLIKA), Recife, Pernambuco Brazil

**Keywords:** *Staphylococcus aureus*, Nasal Colonization, Healthcare Professionals, Bacterial Drug Resistance

## Abstract

Nasal colonization by *Staphylococcus aureus* among healthcare professionals represents a potential risk for the spread of resistant strains in hospital settings. Thus, this cross-sectional study aimed to determine the prevalence of nasal colonization by *S. aureus* and to characterize the antimicrobial resistance profile of the isolated strains from professionals working in a public hospital in Caxias, Maranhão, Brazil. This study was carried out from December 2024 to February 2025. A total of 132 professionals working in a public hospital in the state of Maranhão were included. Among the participants, 36 (27.27%) were colonized by *S. aureus*, and 6 isolates (16.66%) were identified as methicillin-resistant *S. aureus* (MRSA). High resistance rates were observed to penicillin G and ampicillin (94.4%), as well as to the macrolides erythromycin (83.3%) and azithromycin (77.8%). Resistance to tetracycline was detected in 11.1% of the isolates, with 22.2% showing intermediate susceptibility. All isolates were susceptible to clindamycin, chloramphenicol, and rifampicin. Regarding risk factors, working in two healthcare locations compared to one remained significantly associated with nasal colonization after adjustment (OR = 3.15–3.50; *p* < 0.05), whereas longer professional experience was identified as a protective factor (OR ranging from 0.11 to 0.17; *p* < 0.05). These findings demonstrate the circulation of multidrug-resistant *S. aureus* strains among healthcare professionals and highlight the importance of active surveillance, rational antimicrobial use, and strengthened infection control strategies to reduce the spread of resistant pathogens in hospital environments.

## Introduction

 Healthcare-associated infections (HAIs) caused by multidrug-resistant (MDR) microorganisms represent a growing challenge for global public health [1]. Among these pathogens, *Staphylococcus aureus* (*S. aureus*) stands out as one of the main agents involved in surgical site infections, endocarditis, pneumonia, and sepsis [2, 3]. This scenario has been aggravated by the increase in antimicrobial resistance rates presented by this pathogen, especially in methicillin-resistant *S. aureus* (MRSA) strains and MDR strains. Global estimates indicate that, in 2019, MRSA was responsible for more than 100,000 deaths worldwide [4].

Antimicrobial resistance (AMR) in *S. aureus* is a multifactorial phenomenon, resulting from the combination of selective pressure exerted by the extensive use of antimicrobials and the remarkable genetic plasticity of this pathogen [5]. This pathogen has a high capacity to acquire and disseminate resistance determinants through chromosomal mutations and mobile genetic elements, which favors the emergence of multidrug-resistant phenotypes [6–8].

In this context, nasal colonization by *S. aureus* is a silent threat and represents a critical step in the hospital dissemination chain of this pathogen [9]. Colonized healthcare professionals, often asymptomatically, contribute directly to the occurrence of nosocomial infections [10]. This condition is especially concerning due to the microorganism’s ability to remain in the nasal mucosa for long periods, favored by its commensal nature and adaptability to the host [11].

The main route of transmission of *S. aureus* in hospital settings is direct contact between individuals, with healthcare professionals’ hands being particularly important vectors of the pathogen [8]. This dynamic is exacerbated by the high rate of nasal colonization among healthcare workers, as evidenced by studies conducted in different Brazilian institutions, which reinforces the role of these professionals as reservoirs and potential sources of dissemination [12, 13].

Despite the relevance of the topic, there is still a scarcity of research associating the presence of colonizing *S. aureus* with antimicrobial resistance profiles in healthcare professionals in the state of Maranhão. It is worth noting that the region faces a context of economic inequality that directly affects the structure and quality of healthcare services, which underscores the significance of this research gap. Furthermore, antibiotic prescribing remains largely empirical due to the limited standardization of institutional protocols, while the state remains underrepresented in national surveillance data on MDR pathogens. Given this, the present study aimed to determine the prevalence of nasal colonization by *S. aureus* and to characterize the antimicrobial resistance profile of the isolated strains from professionals working in a public hospital in Caxias, Maranhão, Brazil.

## Methods

### Study design, sample collection, and microbiological processing

This cross-sectional, analytical, and exploratory study was conducted between December 2024 and February 2025 at a public hospital located in eastern Maranhão, Brazil. The study population consisted of 132 healthcare professionals from various occupational categories, including nurses (*n* = 35), nursing technicians and assistants (*n* = 54), physicians (*n* = 29), and other professionals (*n* = 14; nutritionists, physiotherapists, pharmacists, and dentists). Participants were characterized using a sociodemographic questionnaire that collected information on age, sex, occupational category, work sector, and length of professional service. Professionals who had received antimicrobial therapy within the two weeks prior to sample collection were excluded.

The sample size was calculated based on a finite population of 300 eligible healthcare professionals. Assuming an unknown prevalence, an expected proportion of 50% (*p* = 0.50) was adopted to ensure maximum sample variability, with a 95% confidence level (Z = 1.96) and a 7% margin of error. The initial estimate was adjusted for a finite population and increased by a 10% safety buffer to account for potential losses or refusals, establishing a minimum required sample of 131 participants. Participants were selected using non-probability convenience sampling. To minimize selection bias inherent to this strategy, recruitment was conducted across different work shifts (day and night) and multiple hospital sectors.

Nasal samples were collected by the principal investigator using sterile swabs, which were rotated thrice against the inner surface of each nostril. Immediately after collection, the swabs were inoculated into Brain-Heart Infusion (BHI) broth and transported under appropriate conditions to the Laboratory of Microbiology and Immunology of Infectious and Parasitic Diseases (LAMIDI/UEMA) within six hours from collection. Cultures were incubated at 35 °C for 18–24 h, then 10 µL was subcultured onto 5% sheep blood agar. Presumptive *S. aureus* colonies, showing β-hemolysis, were subcultured on mannitol salt agar (MSA) and identified via colony morphology, catalase and coagulase tests [14]. Isolates meeting the phenotypic criteria for the presumptive identification of MRSA were subsequently subjected to matrix-assisted laser desorption/ionization time-of-flight mass spectrometry (MALDI-TOF MS) using the Biotyper 3.1 system (Bruker Daltonics, Bremen, Germany) for species-level identification.

### Antimicrobial susceptibility testing and determination of MIC/MBC

Antimicrobial susceptibility testing was performed by the disk diffusion method on Mueller–Hinton agar (MHA; 4 mm depth) using bacterial suspensions adjusted to a 0.5 McFarland standard, according to the Clinical and Laboratory Standards Institute (CLSI, 2025) and Brazilian Committee on Antimicrobial Susceptibility Testing (BrCAST, 2024) guidelines [15, 16]. The following antimicrobial discs were tested at standardized concentrations: cefoxitin (FOX, 30 µg), penicillin G (PEN, 10 U), ampicillin (AMP, 10 µg), azithromycin (AZM, 15 µg), cefazolin (CFZ, 30 µg), erythromycin (ERY, 15 µg), clindamycin (CLI, 2 µg), ciprofloxacin (CIP, 5 µg), gentamicin (GEN, 10 µg), rifampicin (RIF, 5 µg), levofloxacin (LVX, 5 µg), tetracycline (TET, 30 µg), chloramphenicol (CHL, 30 µg), trimethoprim–sulfamethoxazole (SXT, 1.25/23.75 µg), and doxycycline (DOX, 30 µg). Plates were incubated at 35 ± 2 °C for 18–24 h, after which inhibition zone diameters were measured using a digital caliper and interpreted according to CLSI and BrCAST breakpoints. *S. aureus* ATCC 25,923 was included as the quality-control strain.

For oxacillin and vancomycin, minimum inhibitory concentrations (MICs) were determined by broth microdilution in 96-well plates containing Mueller–Hinton broth (MHB) supplemented with serial twofold concentrations of each antimicrobial (0.125–64 µg/mL), yielding a final bacterial inoculum of approximately 5 × 10⁵ CFU/mL per well prepared from a 0.5 McFarland standard suspension. Following incubation at 35 ± 2 °C for 24 h, MICs were determined spectrophotometrically at 620 nm using a Multiskan FC microplate reader (Thermo Scientific) and confirmed visually using 2% resazurin. Resistance phenotypes were classified according to the applicable CLSI and BrCAST criteria, with methicillin-susceptible *S. aureus* (MSSA) or MRSA identified based on an oxacillin MIC ≥ 4 µg/mL and/or a cefoxitin inhibition zone ≤ 21 mm, vancomycin-intermediate *S. aureus* (VISA) defined by a vancomycin MIC of 4–8 µg/mL, and vancomycin-resistant *S. aureus* (VRSA) by a MIC ≥ 16 µg/mL [15]. The minimum bactericidal concentration (MBC) was determined by subculturing aliquots from wells showing no visible growth onto Mueller–Hinton agar, followed by an additional 24 h incubation under the same conditions [17]. These assays were conducted at the Keizo Asami Institute of the Federal University of Pernambuco (iLIKA/UFPE) in independent triplicates.

### Genotypic detection of MRSA and characterization of SCCmec

For DNA extraction, cells cultured in Luria-Bertani broth (LBI) were incubated in lysis buffer containing lysostaphin (200 µg/mL) and lysozyme (20 mg/mL) (Sigma-Aldrich, Saint Louis, MO, USA) for 30 min at 37 °C. Genetic material was then extracted using the QIAamp DNA Mini Kit (QIAGEN, Hilden, Germany), according to the manufacturer’s recommendations. The concentration and purity of the extracts were evaluated using a NanoDrop 2000c spectrophotometer (Thermo Fisher Scientific Inc.), considering the A260/280 ratio for DNA quality verification.

Staphylococcal mec chromosome element (SCCmec) typing of *S. aureus* isolates was performed by multiplex PCR according to the protocol described previously [18] for simultaneous detection of *mecA* gene and SCCmec typing of MRSA isolates. The amplified products were subjected to electrophoresis on a 2% agarose gel, stained with SYBR Safe (Thermo Fisher Scientific Inc.) and subsequently visualized under UV light. This investigation was conducted at the Aggeu Magalhães – Oswaldo Cruz Foundation Pernambuco (IAM/Fiocruz PE).

### Statistical analysis

The analyses were performed using RStudio (2025.05.0). Descriptive analysis included absolute and relative frequencies for qualitative variables and mean, standard deviation, and variance for the quantitative variable. For comparisons between groups, the Chi-square or Fisher’s exact test and the Mann-Whitney U test were applied. Factors associated with *S. aureus* colonization were evaluated by simple and multiple logistic regression, with OR, 95% CI, and *p* < 0.05. Model performance was verified by AIC, Hosmer-Lemeshow test, VIF (< 5), and Likelihood Ratio (LRT). Statistical significance was considered for *p* ≤ 0.05.

### Ethical aspects

The study followed the ethical principles of research involving human subjects and was approved by the Research Ethics Committee of the State University of Maranhão, Brazil (opinion no. 7.156.435; CAAE 83125424.5.0000.5554).

## Results

3.1 Characteristics of the Study Population and *S. aureus* rate Colonization.

A total of 132 professionals were included in the study, representing approximately 44% of the healthcare workforce at the institution during the study period (*n* = 300). From these professionals, 36 *S. aureus* strains were isolated, corresponding to a colonization rate of 27.27% (36/132; 95% CI: 20.40% − 35.43%). The mean age of the participants was 38.94 years (standard deviation ± 10.90), with ages ranging from 24 to 69 years. Women represented the majority of the study population (68.94%). Regarding professional training, nursing technicians or assistants accounted for 40.91% of the participants. In terms of professional experience, 34.09% of participants had between 1 and 5 years of experience, whereas 21.21% had between 6 and 10 years. The apparent prevalence of MRSA among colonized professionals was 16.67% (6/36; 95%CI: 7.87% − 31.89%), while the overall rate, considering all participants in the sample, was 4.54% (6/132; 95%CI: 2.0% − 9.56%). These characteristics are presented in Table 1.

**Table 1:** Social and professional profile and habits of research participants according to colonization by *S. aureus*

	Sample by *S. aureus* colonization		Total Sample (*N* = 132)
	Negative (*n* = 96)	Positive (*n* = 36)	
Age			
Average	40.21	35.79	38.94
Standard Deviation	11.41	8.88	10.9
Variance	130.19	78.85	118.81
	n (%)	n (%)	n (%)
Sex			
Male	27 (28.12%)	14 (38.89%)	41 (31.06%)
Female	69 (71.88%)	22 (61.11%)	91 (68.94%)
Profession			
Nurse	25 (26.04%)	10 (27.78%)	35 (26.52%)
Doctor	17 (17.71%)	12 (33.33%)	29 (21.97%)
Nursing Technician/Assistant	44 (45.83%)	10 (27.78%)	54 (40.91%)
^a^Others	10 (10.42%)	4 (11.11%)	14 (10.61%)
Professional experience			
< 1 year	4 (4.17%)	6 (16.67%)	10 (7.58%)
1 to 5 years	36 (37.5%)	9 (25.00%)	45 (34.09%)
6 to 10 years	17 (17.71%)	11 (30.56%)	28 (21.21%)
11 to 15 years	20 (20.83%)	5 (13.89%)	25 (18.94%)
> 15 years	19 (19.79%)	5 (13.89%)	24 (18.18%)
Smoking			
No	78 (81.25%)	27 (75.00%)	105 (79.55%)
No, but it already happened.	13 (13.54%)	9 (25.00%)	22 (16.67%)
Yes, regularly	2 (2.08%)	0 (0.00%)	2 (1.52%)
Yes, occasionally.	3 (3.12%)	0 (0.00%)	3 (2.27%)

^a^Others: Pharmacists, nutritionists, dentists, and physiotherapists

Among the professionals colonized by *S. aureus*, the occupational profile showed that 52.78% worked in two locations, while 38.89% reported a weekly workload exceeding 40 h. With respect to the work sectors, wards accounted for the highest proportion of colonized professionals (44.44%), followed by intensive care units (36.11%). Regarding the use of personal protective equipment (PPE), 72.22% of colonized professionals reported regular use. The occupational characteristics of the colonized professionals are presented in Table 2.

**Table 2:** Work, exposure, and practices in the work of the research participants

	Sample by *S. aureus* colonization		Total Sample (*N* = 132)
	Negative (*n* = 96)	Positive (*n* = 36)	
	*n* (%)	*n* (%)	*n* (%)
Areas of operation			
One	49 (51.04%)	10 (27.78%)	59 (44.70%)
Two	32 (33.33%)	19 (52.78%)	51 (38.64%)
Three or more	15 (15.62%)	7 (19.44%)	22 (16.67%)
Total weekly workload			
< 20 h	2 (2.08%)	2 (5.56%)	4 (3.03%)
20 to 30 h	20 (20.83%)	7 (19.44%)	27 (20.45%)
31 to 40 h	54 (56.25%)	13 (36.11%)	67 (50.76%)
+ 40 h	20 (20.83%)	14 (38.89%)	34 (25.76%)
Work schedule			
On-call	71 (73.96%)	26 (72.22%)	97 (73.48%)
Housekeeper	7 (7.29%)	1 (2.78%)	8 (6.06%)
Mixed	18 (18.75%)	9 (25%)	27 (20.45%)
Area of activity			
ICU	29 (30.21%)	13 (36.11%)	42 (31.82%)
Infirmary	40 (41.67%)	16 (44.44%)	56 (42.42%)
Surgical center	20 (20.83%)	2 (5.56%)	22 (16.67%)
Ward/Surgical Center	7 (7.29%)	5 (13.89%)	12 (9.09%)
Another area of activity			
Not applicable	48 (50%)	11 (31.58%)	11 (30.56%)
ICU	10 (10.42%)	5 (13.16%)	5 (13.89%)
Infirmary	15 (15.62%)	9 (26.32%)	9 (25%)
Surgical center	12 (12.5%)	4 (10.53%)	4 (11.11%)
^c^Others	11 (11.46%)	7 (18.42%)	7 (19.44%)
Contact with the patient			
Rarely	5 (5.21%)	1 (2.78%)	6 (4.55%)
Weekly	65 (67.71%)	24 (66.67%)	89 (67.42%)
Daily	26 (27.08%)	11 (30.56%)	37 (28.03%)
Use of PPE			
Rarely	15 (15.62%)	1 (2.78%)	16 (12.12%)
Regularly	64 (66.67%)	26 (72.22%)	90 (68.18%)
Occasionally	17 (17.71%)	9 (25%)	26 (19.70%)

^b^Other: Outpatient clinic, clinics, laboratory

3.2 Risk factors for colonization by *S. aureus*.

Comparison tests (Mann-Whitney U, T-test, and chi-square) revealed no statistically significant differences between colonized and non-colonized individuals regarding age and sex (*p* > 0.05), while area of activity (*p* = 0.0507) and weekly workload (*p* = 0.0718) showed marginally non-significant p-values. In simple logistic regression, working in two locations (OR = 2.90; 95% CI: 1.22–7.28; *p* = 0.0181) and a workload exceeding 40 h per week (OR = 2.90; 95% CI: 1.17–7.35; *p* = 0.0219) were associated with higher colonization odds, while professional experience showed an inverse association, with groups of 1–5 years (OR = 0.17; *p* = 0.0162), 11–15 years (OR = 0.17; *p* = 0.0282), and > 15 years (OR = 0.18; *p* = 0.0334) presenting lower colonization probability compared to professionals with less than one year of experience (Table 3). Multiple logistic regression models, built with variables at *p* < 0.20, confirmed working in two locations as an independent risk factor (Model 1: OR = 3.15; 95% CI: 1.13–9.42; *p* = 0.031; Model 2: OR = 3.50; 95% CI: 1.36–9.68; *p* = 0.011) and professional experience as a consistent protective factor across experience groups, while weekly workload did not retain significance after adjustment (Table 4). Among the models tested, Model 2 showed the best performance, evidenced by the lowest AIC (152.5) and the highest significance according to the likelihood ratio test (*p* = 0.013).

**Table 3:** Logistic Regression: Factors Associated with Colonization by *S. aureus*

	I estimated	OR[IC95%]	*p*-value	LRT (*p*-value)
Profession (Ref=Doctor)				0.173
Nurse	-0.568	0.56 [0.19–1.59]	0.2849	
Nursing Technician	-1.1333	0.32 [0.11–0.87]	0.0277*	
Others	-0.568	0.56 [0.13–2.15]	0.4182	
Professional experience (Ref = < 1 year)				0.058 .
1 to 5 years	-1.7918	0.17 [0.03–0.70]	0.0162*	
6 to 10 years	-0.8408	0.43 [0.09–1.85]	0.2639	
11 to 15 years	-1.7918	0.17 [0.03–0.79]	0.0282*	
> 15 years	-1.7405	0.18 [0.03–0.84]	0.0334*	
Number of operating locations (Ref = One location)				0.047*
Two locations	1.06	2.90 [1.22–7.28]	0.0181*	
Three or more locations	0.82	2.28 [0.72–7.05]	0.1499	
Total weekly workload (Ref = 31 to 40 h)				0.1
< 20 h	1,424	4.15 [0.46–37.38]	0.1736	
21 to 30 h	0.3742	1.45 [0.48–4.09]	0.4858	
+ 40 h	1.0674	2.90 [1.17–7.35]	0.0219*	

reference category; *: *p*-value significant with 95% confidence; Others: Pharmacists, dentists, nutritionists and physiotherapists; *LRT* Likelihood Ratio Test

**Table 4:** Multiple Logistic Regression: Factors Associated with *S.* *aureus* Colonization

Variables included in the models	Model 1		Model 2	
	OR[IC95%]	*p*-value	OR[IC95%]	_*p*−value_
Number of operating locations (Ref = One location)				
Two locations	3.15[1.13–9.42]	0.031*	3.50 [1.36–9.68]	0.011*
Three or more locations	1.80 [0.78–7.20]	0.417	2.67 [0.75–9.55]	0.124
Professional experience (Ref = < 1 year)				
1 to 5 years	0.17[0.03–0.79]	0.026*	0.14 [0.03–0.65]	0.013*
6 to 10 years	0.30[0.05–1.49]	0.147	0.29 [0.05–1.37]	0.127
11 to 15 years	0.11[0.02–0.63]	0.016*	0.1 [0.01–0.54]	0.009**
> 15 years	0.12[0.02–0.71]	0.023*	0.12 [0.02–0.64]	0.016*
Weekly workload (Ref = 31 to 40 h)				
< 20 h	2.92[0.24–32.6]	0.367	-	_−_
20 to 30 h	1.22[0.36–3.85]	0.736	-	_−_
+ 40 h	2.37[0.81–7.16]	0.118	-	_−_
Model evaluation criteria				
AIC	155.3		152.5	
VIF	< 5		< 5	
LRT (p-value)	0.022*		0.013*	
HLT (p-value)	0.311		0.212	

reference category; *: *p*-value significant with 95% confidence; *AIC* Akaike Evaluation Criterion, *VIF* Variance Inflation Factor, *LRT* Likelihood Ratio Test, *HLT* Hosmer-Lemeshow Test; Model 1: Colonization ~ location quantity + experience time + experience; Model 2: Colonization ~ location quantity + experience time

### Antimicrobial susceptibility and determinant genetic resistance

A total of six *S. aureus* isolates exhibited an oxacillin MIC ≥ 4 µg/mL and were classified as MRSA, whereas 30 exhibited an MIC < 4 µg/mL and were classified as MSSA according to CLSI (2025) guidelines. MIC and MBC values for vancomycin remained within the sensitivity range for both groups, with no evidence of VISA or VRSA phenotypes. These results are detailed in Table 5.

**Table 5:** Phenotypic resistance profile of *S. aureus* strains according to MIC and MBC for oxacillin and vancomycin

STRAINS	OXA		VAN	
	MIC	MBC	MIC	MBC
µg/mL				
P01	0,125	0,125	2	2
P03	0,250	0,5	2	2
P05	0,250	0,5	2	2
P13	0,125	0,5	2	4
P17	0,5	1	2	4
P18	0,250	1	2	2
P20	0,250	0,5	2	2
P23	2	4	2	2
P30	0,125	0,5	2	2
P37	0,250	0,5	2	2
P64	0,5	1	2	4
*P70	4	16	1	1
P73	2	4	2	2
P74	1	4	2	2
P78	0,125	0,250	1	2
P79	0,125	0,250	2	2
P81	0,5	1	2	2
*P84	8	8	2	4
*P85	16	16	2	2
P86	0.5	0,5	0,5	0,5
P90	0,125	0,5	0,125	0,5
P91	0,125	0,5	0,125	0,5
P97	2	8	2	8
P102	1	2	1	2
*P103	8	16	1	1
*P104	8	32	2	4
P108	0,5	1	0,5	1
P109	0,125	0,125	0,125	0,125
P115	1	1	1	1
P121	2	2	2	2
P124	1	2	1	2
P125	0,125	0,5	0,125	0,125
*P126	4	8	1	2
P129	0,125	0,125	0,5	0,5
P130	0,125	0,5	0,125	0,5
P132	2	8	2	8

*MIC* Minimum Inhibitory Concentration, *MBC* Minimum Bactericidal Concentration,* OXA* Oxacillin, *VAN *Vancomycin. *: Strains classified as MRSA according to CLSI (2025) and BrCAST (2024) guidelines; CI; P: Identification code of the clinical isolated *Staphylococcus areus* strain corresponding to the study participant

**Fig. 1:**
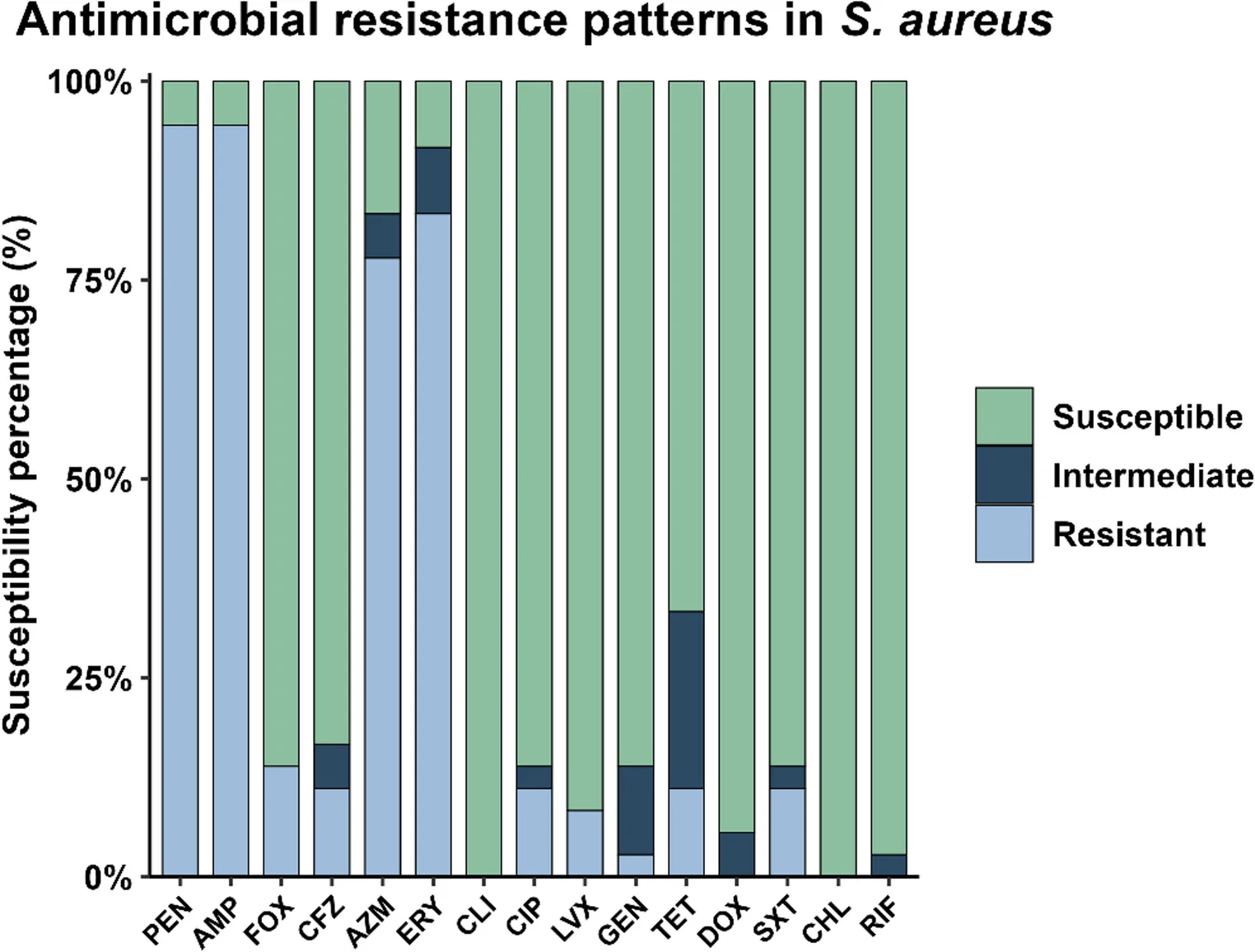
Antimicrobial resistance and susceptibility patterns of *Staphylococcus aureus* isolates according to the tested antibiotics. PEN: Penicillin G; AMP: Ampicillin; FOX: Cefoxitin; CFZ: Cefazolin; AZM: Azithromycin; ERY: Erythromycin; CLI: Clindamycin; CIP: Ciprofloxacin; LVX: Levofloxacin; GEN: Gentamicin; TET: Tetracycline; DOX: Doxycycline; SXT: Trimethoprim–Sulfamethoxazole; CHL: Chloramphenicol; RIF: Rifampicin

Eighteen strains (50%; 95%CI: 34.47% − 65.53%) among 36 strains isolated from professionals showed multidrug resistance (MDR). The highest resistance rates were observed for the β-lactams penicillin G and ampicillin, both with 94.44% (95%CI: 81.86% − 98.46%) of isolates classified as resistant. Among macrolides, resistance was high for erythromycin (83.33%; 95%CI: 68.11% − 92.13%) and azithromycin (77.78%; 95%CI: 61.91% − 88.28%). Tetracycline resistance was observed in 11.11% (95%CI: 4.41% − 25.31%) of isolates, while 22.22% (95%CI: 11.72% − 38.08%) showed an intermediate profile. No resistance to doxycycline was detected (95%CI: 0.00% − 9.64%). For fluoroquinolones, resistance rates were 11.11% (95%CI: 4.41% − 25.31%) for ciprofloxacin and 8.33% (95%CI: 2.87% − 21.83%) for levofloxacin. All strains were susceptible to clindamycin, chloramphenicol and rifampicin (95%CI: 0.00% − 9.64%). However, as the D-test was not performed, inducible clindamycin resistance cannot be excluded. Cefoxitin resistance was identified in 16.67% (95%CI: 7.87% − 31.89%) of isolates, consistent with the proportion of MRSA strains detected in the study (Fig. 1).

**Fig. 2:**
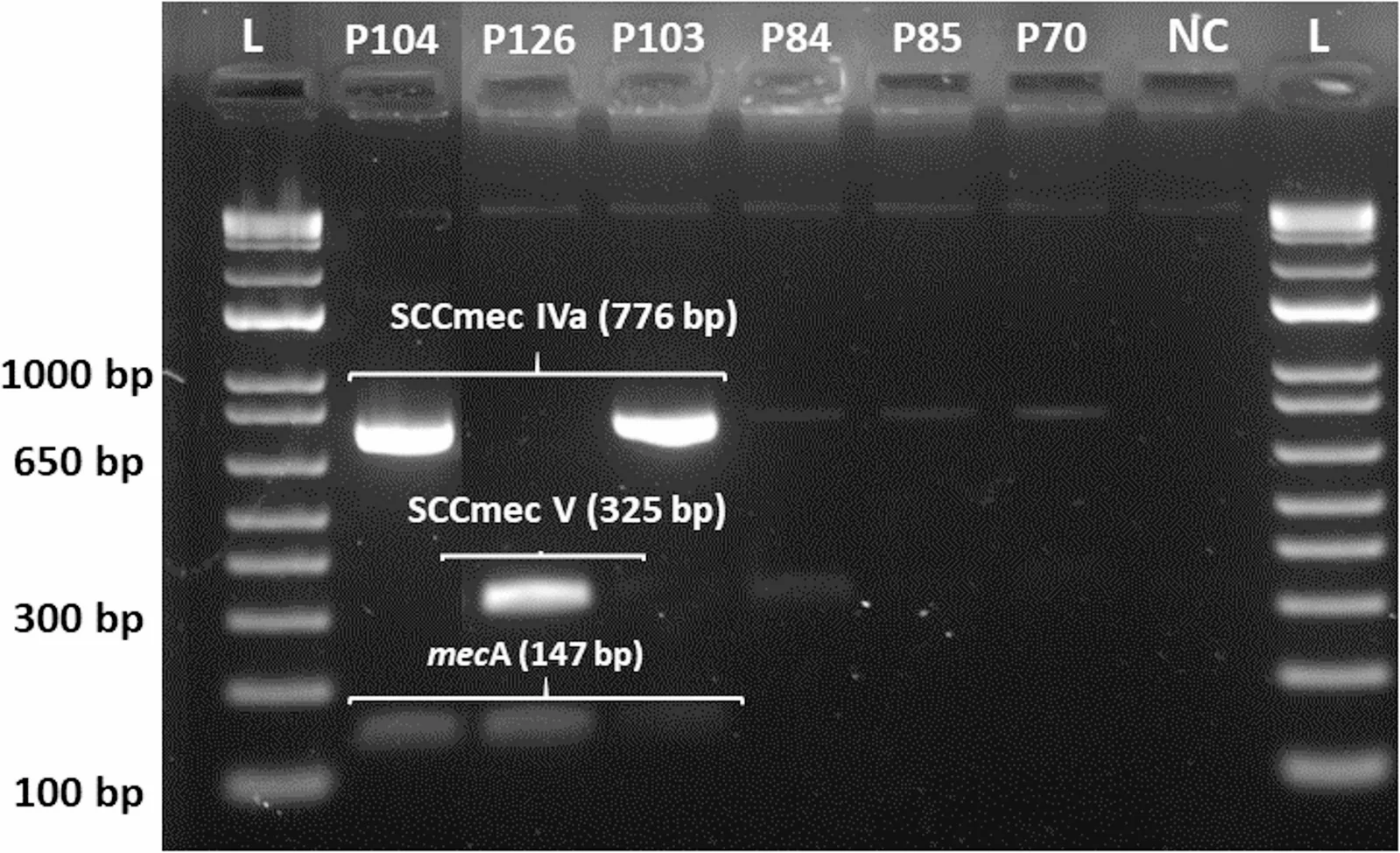
Molecular characterization and SCCmec typing of MRSA isolates by multiplex PCR. A multiplex PCR assay was performed using multiple primer pairs targeting SCCmec types I, II, III, IVa, IVb, IVc, IVd, and V, together with primers targeting the *mecA* gene. Isolates P104 and P103 yielded an approximately 776-bp amplicon corresponding to the SCCmec IVa target, whereas P126 yielded an approximately 325-bp amplicon corresponding to the SCCmec V target. P104, P126, and P103 also yielded the expected approximately 147-bp *mecA* amplicon. No amplification corresponding to any of the tested SCCmec types or *mecA* was detected in isolates P84, P85, or P70. The negative control (NC) showed no amplification. L, 100-bp DNA molecular size marker. The multiplex PCR amplification profiles were used to assign the SCCmec type of the positive isolates

Six *S. aureus* strains phenotypically classified as MRSA were subjected to molecular analysis by PCR for detection of the *mecA* gene and SCCmec typing. Three of the six strains amplified the *mecA* gene (147 bp) and were characterized as either SCCmec IVa (776 bp) or SCCmec V (325 bp) (Fig. 2). The same six strains identified as MRSA were confirmed as *S. aureus* by MALDI-TOF MS, with identification scores between 2.14 and 2.34, and presented spectrometric subtyping classified as “presumptive PSM positive MRSA”, indicating protein profiles associated with the methicillin resistance phenotype.

## Discussion

The results indicate the presence of MRSA strains in the study population. Among the isolates, half of the strains presented an MDR pattern. The highest resistance rates observed were for the β-lactam and macrolide classes. Similar results were reported in other studies where MDR strains of *S. aureus*, with high rates of β-lactam resistance [19–21].

In addition to resistance to the aforementioned drugs, another relevant finding was resistance to tetracycline (11.11%, with a further 22.22% showing intermediate susceptibility). The pattern observed for this class follows reports of a progressive increase in this resistance over the last decade [22] frequently associated with multidrug resistance profiles and a significant reduction in available therapeutic options [23].

Resistance rates to gentamicin and fluoroquinolones were lower, suggesting that these classes remain potentially therapeutic in the analyzed scenario. All isolates remained susceptible to chloramphenicol and clindamycin; nevertheless, as the D-test was not conducted, the presence of inducible clindamycin resistance cannot be entirely ruled out. The high sensitivity observed for these antibiotics may be related to their infrequent use in local clinical practice, either due to higher cost or limited availability, which reduces selective pressure on circulating strains [20].

The MRSA strains analyzed in this study exhibited varied genotypic profiles: three of the six isolates (P104, P103 and P126) carried the *mecA* gene detected by PCR and belonged to the staphylococcal cassette subtypes SCCmec IVa and V, respectively. These mobile elements that confer methicillin resistance by encoding PBP2a are frequently associated with MRSA lineages with a greater capacity for dissemination in the community and hospitals, contributing to the complexity of resistance in different clinical contexts [24, 25]. The remaining strains (P84, P85 and P70) did not amplify the *mecA* gene, suggesting alternative resistance mechanisms, such as hyperproduction of β-lactamases or mutations in the pbp4 promoter region or in gdpP, which lead to PBP4 overproduction and increased cell wall cross-linking, which require further molecular investigation [26–28].

Recent studies combining molecular typing techniques with initial identification by MALDI-TOF MS have reinforced the usefulness of this method in the rapid recognition of *S. aureus* and in the inclusion of resistance profiles associated with different SCCmec, which assists both in epidemiological surveillance and in the prediction of resistance patterns (e.g., types II and IVa) from specific proteomic signatures — an approach that has shown promise for preliminary classification of MRSA clones in clinical routines [29, 30].

The overall *S. aureus* colonization rate in this study (27.27%) aligns with reports from Ecuador (23.7%) and Oman (20.5%) [31, 32], yet remains lower than rates documented in southern (42.9%) and northeastern (75%) Brazil [13, 33]. Similarly, our overall MRSA prevalence (4.64%) was comparable to findings from India (6.33%) but lower than those from the Philippines (13%) [34, 35]. Among *S. aureus* carriers, MRSA accounted for 16.66%, falling below the global pooled estimate of 32.8% for healthcare workers [36]. These disparities likely stem from regional differences in study populations, healthcare infrastructure, infection control adherence, local antimicrobial pressure, and microbiological surveillance capacity.

The presence of asymptomatic *S. aureus* favors the maintenance of MDR strains in the hospital environment, since healthy carriers act as silent reservoirs capable of sustaining the circulation of the pathogen among professionals and patients [37–39].

Similar results were observed by Dobrachinski et al. [19] in a study conducted in Brazil with 445 health professionals in a public service in the state of Bahia, in which the nasal colonization rate by *S. aureus* was 39.5%, and 32.3% of the strains were identified as MRSA. These data corroborate the significant occurrence of resistant strains in hospital environments, pointing to a scenario that indicates that the circulation of MRSA is a present and underdiagnosed reality in different regions of the country.

The variations in prevalence rates observed between different studies may be related to multiple factors, such as socioeconomic inequalities, especially in developing countries [40]. Organizational problems such as weak public policies aimed at microbiological surveillance and the rational use of antimicrobials directly affect the effectiveness of contingency actions for the spread of this pathogen [41, 42].

It is worth mentioning the scarcity of recent Brazilian investigations evaluating nasal colonization by *S. aureus* in healthcare professionals, especially with an active screening approach. Thus, this absence of new data in the subsequent period reinforces the importance and contribution of this work to fill this epidemiological gap. In the state of Maranhão, MRSA notifications related to healthcare are reported, but there is still underreporting and a lack of structured surveillance programs. The identification of asymptomatic carriers, as evidenced in this study, can support regional monitoring and prevention actions, contributing to reducing the spread of resistant pathogens in the state.

In our analyses, age was not associated with *S. aureus* colonization. However, studies indicate a higher risk in older professionals due to longer occupational exposure time [10, 30, 43], while other investigations identify a higher prevalence among younger professionals, attributed to less familiarity with infection control practices and less clinical experience [21, 43]. The absence of association observed in our sample may reflect a relatively homogeneous age distribution or particularities of the local work dynamics that mitigate inequalities in exposure between age groups.

Professional experience was the factor most consistently associated with a lower probability of colonization. In the adjusted models, professionals with 1 to 5 years (OR = 0.17; *p* = 0.026), 11 to 15 years (OR = 0.11; *p* = 0.016), and more than 15 years of experience (OR = 0.12; *p* = 0.023) showed a significant reduction in risk when compared to the group with less than 1 year of experience. This protective effect is consistent with findings from different contexts, in which more experienced professionals demonstrate greater mastery of institutional protocols, greater adherence to biosafety practices, and greater familiarity with contamination prevention strategies [44, 45].

Although the professional category did not show a statistical association (*p* > 0.05), a marked difference was observed in the frequency distribution between the classes. Physicians concentrated the highest proportion of colonized professionals (33.33%), followed by nursing technicians/assistants and nurses (27.78% for both). In addition, surgical and postoperative wards had the highest proportion of colonized individuals (44.44%), a finding similar to that reported by Giri et al. [46], who identified a high prevalence in these sectors. Although these differences were not statistically confirmed, they reflect areas and professional categories with greater intensity of care contact and movement between different clinical environments, which may influence exposure to resistant microorganisms [47, 48].

The higher concentration of colonized professionals among physicians may be related to their greater mobility between sectors, multiple working positions, and longer weekly workloads — factors reported by this category in our sample — as well as frequent direct contact with patients. Although not statistically significant in our analysis, similar patterns were described in Somalia, Ethiopia, and Nepal, where physicians were also the predominant colonized category (17.6%, 11%, and 11.4%, respectively) [20, 46, 49]. Corroborating these data, a Brazilian study found 76.6% of MRSA strains isolated from physicians professionals’ noses, hands, and lab coats associated with those who had direct patient contact [33].

Although working over 40 h per week was associated with colonization in the unadjusted analysis (OR = 2.90; 95% CI: 1.17–7.35; *p* = 0.0219), this association did not persist in multivariate modeling. Nonetheless, prolonged shifts have been recognized as potential facilitators of colonization, as documented in Libyan hospitals where working 18–24 h daily yielded higher colonization rates (59.3%; *p* = 0.010), likely due to fatigue-related declines in biosafety compliance [50]. Conversely, holding multiple jobs remained significantly associated with *S. aureus* detection across adjusted models (Model 1: OR = 3.15; 95% CI: 1.13–9.42; *p* = 0.031; Model 2: OR = 3.50; 95% CI: 1.36–9.68; *p* = 0.011). This finding reinforces that professional mobility between distinct healthcare environments exposes workers to diverse microbiological ecosystems, facilitating cross-contamination and the institutional dissemination of resistant strains [51].

The presence of professionals colonized by MDR strains reinforces the role of the hospital environment as an important locus for the maintenance and amplification of antimicrobial resistance [52]. The constant circulation of these microorganisms increases selective pressure and favors the persistence of resistant clones, directly contributing to the advancement of AMR in the local context [53].

These findings underscore the need for active screening among healthcare workers, enhanced hand hygiene, and structured infection control strategies. In practice, hospital surveillance should prioritize high-risk units — such as intensive care and surgical wards—using periodic nasal swabbing, particularly during outbreak investigations [53]. Furthermore, managing colonized personnel may include temporary reassignment or targeted decolonization protocols (e.g., intranasal mupirocin and chlorhexidine body washes) with subsequent confirmatory cultures [54]. To our knowledge, this is the first study to document MDR and MRSA nasal colonization among healthcare workers in eastern Maranhão, Brazil, emphasizing the critical role of local epidemiological surveillance and antimicrobial stewardship in this region.

It is important to note that this study was conducted at a single healthcare institution, and the characteristics of its healthcare workforce may differ from those of healthcare professionals in other institutions, regions, or healthcare settings. Since nasal samples were only collected once, long-term carrier status could not be determined, suggesting future studies should involve repeated sampling.

## Conclusion

The susceptibility profile of the isolated *S. aureus* strains revealed the presence of multidrug-resistance, including MRSA phenotypes, with high rates of resistance to macrolides, β-lactams, and tetracyclines, indicating a significant limitation of the therapeutic options available in the evaluated hospital setting. These findings reinforce the relevance of antimicrobial resistance as a persistent challenge in clinical practice. Furthermore, the identification of asymptomatic carriers - with employment at multiple institutions identified as an independent risk factor - emphasizes the urgent need for active surveillance and targeted infection control strategies to mitigate the spread of resistant strains in healthcare settings.
